# Bacterial extracellular vesicles at the interface of gut microbiota and immunity

**DOI:** 10.1080/19490976.2024.2396494

**Published:** 2024-09-28

**Authors:** Inês Melo-Marques, Sandra Morais Cardoso, Nuno Empadinhas

**Affiliations:** a CNC-UC - Center for Neuroscience and Cell Biology, University of Coimbra, Coimbra, Portugal; b CIBB - Centre for Innovative Biomedicine and Biotechnology, University of Coimbra, Coimbra, Portugal; c Institute of Cellular and Molecular Biology, Faculty of Medicine, University of Coimbra, Coimbra, Portugal

**Keywords:** Bacterial extracellular vesicles, gut microbiota, gut immunity, gut dysbiosis, colonization resistance

## Abstract

Bacterial extracellular vesicles (BEVs) are nano-sized lipid-shielded structures released by bacteria and that play an important role in intercellular communication. Their broad taxonomic origins and varying cargo compositions suggest their active participation in significant biological mechanisms. Specifically, they are involved in directly modulating microbial ecosystems, competing with other organisms, contributing to pathogenicity, and influencing the immunity of their hosts. This review examines the mechanisms that underlie the modulatory effects of BEVs on gut dynamics and immunity. Understanding how BEVs modulate microbiota composition and functional imbalances is crucial, as gut dysbiosis is implicated not only in the pathogenesis of various gastrointestinal, metabolic, and neurological diseases, but also in reducing resistance to colonization by enteric pathogens, which is particularly concerning given the current antimicrobial resistance crisis. This review summarizes recent advancements in the field of BEVs to encourage further research into these enigmatic entities. This will facilitate a better understanding of intra- and interkingdom communication phenomena and reveal promising therapeutic approaches.

## GRAPHICAL ABSTRACT

**Figure:**
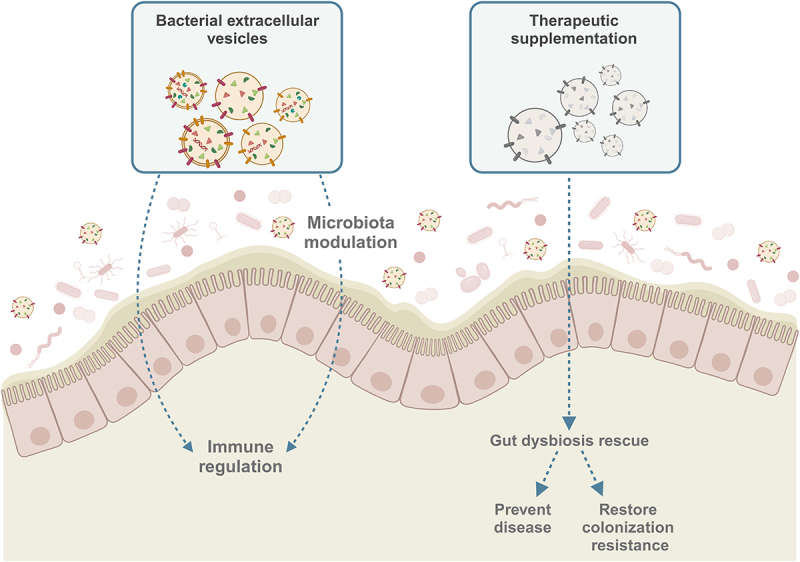

## 1. Introduction

Extracellular vesicles (EVs) can be secreted by cells belonging to all three domains of life: Bacteria, Archaea and Eukarya.^1^ Although eukaryotic EVs have been extensively studied for many years,^2^ interest in EVs produced by Bacteria and Archaea has only recently increased, given their growing biomedical potential, both as diagnostic tools and therapeutic agents themselves, but also as platforms for drug delivery and vaccine development with possible biomedical engineering applications.^3^ Bacterial extracellular vesicles (BEVs) were first identified in *Escherichia coli* in 1966^4^ and in *Vibrio cholerae* in 1967.^5^ Further research has shown that these vesicles can be secreted by members of the human gut microbiota and help to regulate microbial interactions in the gastrointestinal milieu, while in association with host cells and interacting with the host immune and enteric nervous systems.^6–8^ Their functions in this context, range from nutrient acquisition to microbial competition and protection against toxins and antibiotics.^9^ Numerous studies have shown that BEVs play a role in various diseases characterized by an impaired gut microbiota, also known as gut dysbiosis, including gastrointestinal,^10–13^ metabolic,^14^ and neurodegenerative disorders.^15^ The use of BEVs have indeed been proposed as an alternative therapeutic approach and have shown promising results for models of intestinal inflammation.^10–13^ BEVs have clear immunomodulatory effects, depending on their specific cargo and interaction with target cells.^16,17^ Given that microbiome-associated diseases exhibit marked immune dysregulation,^18^ the study of BEVs as modulators of gut disease should be explored in relation to gut immunity. Shedding light on this microbiota-gut immunity axis in the context of BEVs is essential to fully understand vesicle biology and its implications in disease, while dissecting the best ways to devise and modulate their potential applications.

This review focuses on BEVs, specifically in the context of the human gut microbiota. It discusses how BEVs can simultaneously modulate gut microbiota populations and host immunity, and explores their therapeutic potential to rescue dysbiotic gut ecosystems. Finally, we address the urgency of the antimicrobial resistance (AMR) crisis and discuss the dual role of BEVs in colonization resistance (CR), supported by their immunological properties.

## 2. Extracellular vesicle biology

### 2.1. Extracellular vesicles classification and function

**Figure 1.**
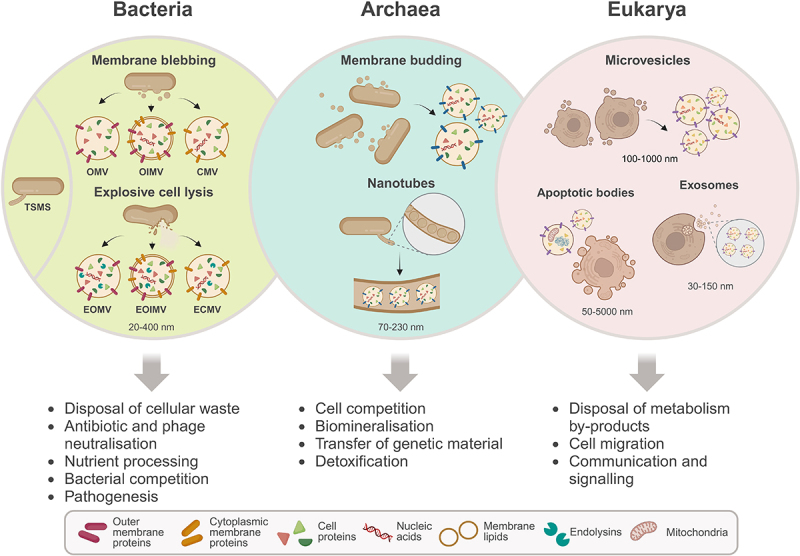
Diversity of extracellular vesicles in the three domains of life.

Extracellular vesicles, which essentially consist of a circular lipid layer surrounding specific cargo molecules, can be categorized according to the organism from which they originate (Figure 1).^1,19^ Eukaryotic vesicles can be classified into three main groups: cellular microvesicles, also known as microparticles or ectosomes, apoptotic bodies and exosomes.^20,21^ Microvesicles, with an average size between 100 and 1000 nm are shed by continuous membrane budding, with various functions regarding cell-cell communication, by transporting different cargo molecules, such as hormones and growth factors but also immune mediators. Apoptotic bodies, which are secreted upon the process of apoptosis, have a size range of 50 to 5000 nm, are formed in response to changes in membrane dynamics resulting from the cell death process and can contain different cellular proteins but also larger structures such as entire organelles and chromatin. Finally, exosomes range in size from 30 to 150 nm, and originate from exocytosis by fusion between the plasma membrane and that of multivesicular endosomes. Their functions are context-dependent and range from the disposal of cell metabolism by-products to cell migration and communication and are thus involved in processes such as embryonic development, regulation of immune responses and disease progression.^20,21^ Archaea-derived vesicles, whose size range is not yet fully defined, but estimated to be between 70 and 230 nm,^22,23^ can be produced by membrane budding or in the form of tubular structures called nanotubes, composed of a number of discrete aligned EVs covered in a unifying S-layer.^24^ These vesicles have been implicated in cell competition,^22^ biomineralisation,^25^ transfer of genetic material^26^ and detoxification.^27^

Bacterial extracellular vesicles can be produced by membrane budding, forming outer-membrane vesicles (OVM) and outer-inner membrane vesicles (OIMV), produced by Gram-negative bacteria, and cytoplasmic membrane vesicles (CMV), produced by Gram-positive strains. Explosive cell lysis and reassembly of fragments also produces each of these vesicle types, EMOV, EOIMV and ECMV, respectively. In Bacteria, vesicles can also be produced in the form of tube-shaped membranous structures (TSMS). Archaea produce membrane vesicles by budding or in the form of nanotubes containing chains of vesicles covered in a protective layer. Eukaryotic vesicles include microvesicles, produced by membrane budding, apoptotic bodies, produced during apoptosis and exosomes, derived from the endocytic pathway. Depending on the domain, extracellular vesicles can have a variety of functions. (Created with BioRender.com).

In the case of BEVs, with diameters between 20 and 400 nm, the classification is more complex due to the variability of membrane organizations, depending on the presence or absence of a cell wall and the mechanism of vesicle production.^28^ Accordingly, vesicles originating from membrane blebbing (B-type) include outer membrane vesicles (OMV), outer-inner membrane vesicles (OIMV), both of which are produced by Gram-negative bacteria, and cytoplasmic membrane vesicles (CMV), which are produced by Gram-positive bacteria. Alternatively, vesicles can result from explosive cell lysis (E-type) mediated by phage-derived endolysins or autolysins and followed by reassembly of membrane fragments. This method of vesicle formation was first discovered in *Pseudomonas aeruginosa* and later found apply to vesicles with any of the different membrane types.^29^ This category includes explosive outer membrane vesicles (EOMV), explosive outer-inner membrane vesicles (EOIMV), these two in the case of Gram-negative bacteria, or CMV again in Gram-positive, but in this case derived from bubbling cell death, called explosive cytoplasmic membrane vesicles (ECMV).^9,28^ Both Gram-positive and Gram-negative bacteria can also produce tube-shaped membranous structures (TSMS), which include nanotubes, nanowires and nanopods.^30,31^ Nanotubes can be simple tubular structures or alternatively display consecutive constrictions, and both of which have a shared lumen. Nanopods consist of numerous aligned EV, that can have electrical properties, in which case are called nanowires, that often acquire a smooth filamentous structure.^1,28^ Throughout this review, the different types of bacterial vesicles will be collectively defined as BEVs, where appropriate, according to the updated nomenclature guidelines.^19^

### 2.2. Vesicle cargo and observed physiological consequences

Membrane vesicles can have a variety of contents, depending on the mechanism of formation and also on the physiological state of the cell. OMV, derived from membrane blebbing, consist mainly of periplasmic proteins, which may include virulence factors, and outer membrane components. OIMV, also originating from blebbing, are surrounded by outer and inner membrane lipids and therefore contain periplasmic but also cytoplasmic contents, including various proteins, nucleic acids and other cytosolic molecules such as ATP. This is similar to CMV, which originate from Gram-positive bacteria and therefore have only a single membrane. Vesicles resulting from explosive cell lysis can contain a variety of components, from proteins of different origins, to nucleic acids and even phages and endolysins.^1,9,28^ In the case of *Pseudomonas aeruginosa*, DNA was also found attached to the vesicle membrane following cell lysis, a localization that was found to be more common than inside the vesicle.^32^

Interestingly, the composition of the BEVs cargo determines the range of functions these vesicles can perform. For example, vesicles can be used for disposal of cellular waste, such as protein accumulation under stress conditions, which can then eliminated in the fecal material.^33^ Considering their similarity to the plasma membrane, BEVs can be used to neutralize antibiotics or other membrane targeting compounds and phages, thus minimizing their interaction with the parent cell.^34,35^ Similarly, BEVs can also carry β-lactamase and directly degrade antibiotics to prevent them from reaching the target cell.^36^ Other functions include nutrient acquisition and processing by hydrolytic enzymes,^37^ bacterial competition including by carrying bioactive molecules,^38^ and pathogenesis by delivering an array of virulence factors and toxins to host cells, while also interacting with different immune cell types to modulate their function.^6,7^ Considering that BEVs release can be constitutive or be induced following cell stress, like iron limitation, pH, exposure to antibiotics or other peptidoglycan-degrading enzymes, integration of hydrophobic compounds into the plasma membrane, among others, vesicle cargo and functions will be extremely variable and often show changes over time.^28^ In addition, several mutations affecting membrane function, for example in the genes *vacJ* and *yrb* encoding phospholipid transporter components,^39^ or in the membrane integrity protein OmpA, can similarly lead to an overvesiculating phenotype.^40^ Antibiotic treatment and reactive oxygen burst induced by H_2_O_2_ can also increase BEVs secretion, including the presence of β-lactam-degrading enzymes in those vesicles.^41,42^ The specific class of antibiotic used can also have an effect on BEVs cargo. Specifically, ciprofloxacin was found to upregulate Shiga toxin 2a in *Escherichia coli* membrane vesicles.^43^ In addition, the composition of the cargo, analyzed by SDS-PAGE, and the biological properties were found to be dependent on the composition and supplementation of culture media.^44^

Differences in vesicle cargo can also affect their interaction with the host. BEVs from *Bifidobacterium longum* OA44, which showed anti-inflammatory effects *in vitro*, were enriched in proteins related to metabolic pathways, ribosomes and secondary metabolite biosynthesis. Other relevant categories included ABC transporters and quorum sensing molecules.^45^ Another study, using BEVs derived from *Bacteroides thetaiotaomicron*, identified enrichment in a number of proteins, in particular dipeptidyl peptidases, an asparaginase and a bile salt hydrolase that was shown to be capable of degrading bile acids.^46^

Although the regulation of cargo packaging in BEVs is not yet fully understood, comparative proteomic studies using *Bacteroides* have shown that cargo content is adapted to nutrient availability by selectively packaging the enzymes needed to degrade the specific polysaccharide or glycosaminoglycan supplemented in the minimal media.^47^ The same study also showed that cargo packaging is indeed a selective process, revealing that the presence of a negatively charged N-terminal motif can selectively sort lipoproteins into outer membrane vesicles.^47^

## 3. The interplay between gut microbiota and the immune system

### 3.1. Gut microbiota overview

**Figure 2.**
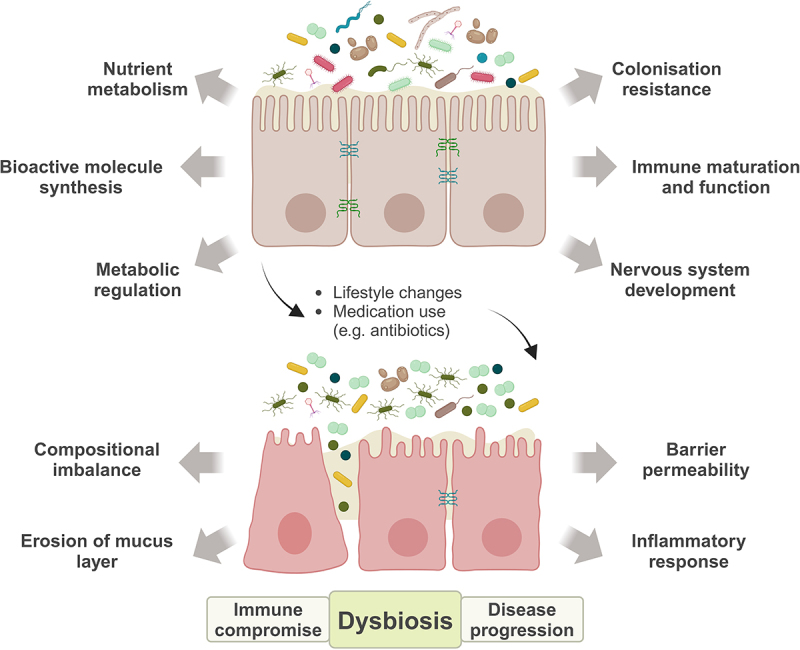
Microbiota in health, dysbiosis and disease.

The collection of microorganisms including bacteria, archaea, fungi, viruses and microeukaryotes that inhabit the human body can be collectively defined as the human microbiota.^48^ This group of microorganisms can be categorized according to where they are found in the body, namely the skin and the gastrointestinal (GI), respiratory and urogenital tracts.^48^ A proper balance between all members of the microbiota is essential to ensure homeostasis,^49^ given their involvement in various physiological processes, including nutrient metabolism and the synthesis of bioactive molecules, such as short-chain fatty acids (SCFAs), vitamins, hormones and even neurotransmitters or their precursors, among other functions (Figure 2).^50^ The microbiota also confer resistance to enteric pathogens through direct and indirect mechanisms of colonization resistance.^51^ It also promotes metabolic regulation^52^ and plays a role in the development of the innate and adaptive immune^18^ and nervous systems.^53^ Similarly, perturbations to the composition of the microbiota or its network of interactions can have strong and lasting consequences, capable of triggering a variety of pathological conditions.^54^ The use of antibiotics has been linked to various diseases not only of the gastrointestinal tract, namely inflammatory bowel disease (IBD)^55^ and loss of colonization resistance,^56^ but also affecting other body areas, being associated with oncological,^57^ autoimmune^58^ and even neurodegenerative diseases.^59,60^ By altering the gut microbial composition, specifically by decreasing the abundance of several key commensal populations, we observe shifts to an alternative network of dynamics. Increased abundance of previously low abundance species becomes difficult to counteract and tends to persist over time.^61,62^ In addition, such changes are accompanied by a marked immune dysregulation in the gut, characterized by an inflammatory environment marked by immune cell activation and the consequent production of pro-inflammatory cytokines, such as IL-1β and IL-18.^63^

The human gut microbiome offers a set of protective functions that depend on its stable composition and dynamics. Events that trigger dysbiosis can compromise the normal function of the microbiota and promote the depletion of protective taxa while increasing opportunistic members. This can lead to gastrointestinal, metabolic and neurological disorders accompanied by immune dysregulation. (Created with BioRender.com).

### 3.2. Gut structured immunity and commensal interactions

**Figure 3.**
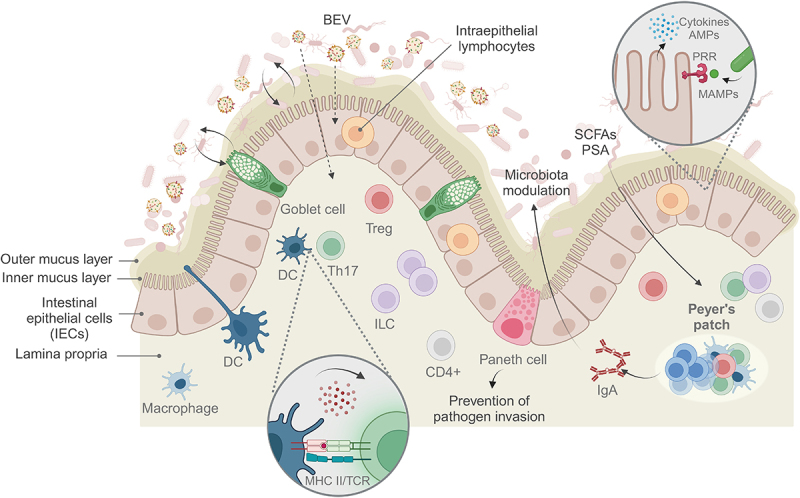
Gut immune cells homeostasis.

The microbiome composition and functions are intrinsically linked to immune regulation, acting in a bidirectional manner, as demonstrated in germ-free (GF) animals, which lack a gut microbiota and are severely immunocompromised.^64^ Specific and targeted immune activation is required to maintain a stable microbiota composition, enriched in commensals and able to respond rapidly to exogenous microbes (Figure 3). Conversely, immune imbalances in the gut, caused by genetic alterations or changes in the composition of the microbiota, may create opportunities to further reinforce microbiota imbalances, which can favor pathogen colonization.^18,65,66^ In addition, altered gut immune activation has been implicated in a variety of bowel diseases, including ulcerative colitis (UC) and Crohn’s disease, as well as in systemic immune dysregulation, with consequences in other body sites.^18^

The gut immune system offers a first layer of protection against exogenous agents and includes the outer and inner mucus layers, the epithelial layer, with goblet cells, Paneth cells and intraepithelial lymphocytes, and the lamina propria, where different types of immune cells can be found. Peyer’s patches can also be found in this tissue and consist of specialized lymphoid structures where B cell maturation takes place. BEVs, bacterial extracellular vesicles; DC, dendritic cell; Th17, T helper 17 cell; ILC, innate lymphoid cell; MHC II, major histocompatibility complex class II; TCR, T cell receptor; Treg, regulatory T cell; SCFAs, short-chain fatty acids; PSA, polysaccharide A; AMPs, antimicrobial peptides; PRR, pattern recognition receptor; MAMPs, microbe-associated molecular pattern. (Created with BioRender.com).

The intestinal barrier is considered to be the first layer of protection against exogenous substances and invading pathogens. This barrier separates the intestinal lumen from the internal environment and starting from the lumen consists of the outer and inner mucus layers, followed by the intestinal epithelial cell (IEC) layer, and then the lamina propria, which contains diverse immune cell types, and below which is the submucosal layer, where Peyer’s patches can be found.^14^ The mucus layer is composed of mucin, secreted by goblet cells and is in close interaction with the gut microbiome, which helps to maintain its optimal thickness.^67^

IEC form a continuous protective layer of tightly associated cells via their tight junctions and can be regulated by interactions with commensal bacteria.^14^ For example, butyrate production by some members of the gut microbiota was found to modulate IEC function, by stabilizing the transcription factor hypoxia-inducible factor (HIF) to maintain barrier integrity.^68^ More specifically, butyrate produced by *Faecalibacterium prausnitzii* was shown to interact with HIF and promote IEC secretion of IL-18, an anti-inflammatory cytokine implicated in the promotion of intestinal barrier integrity.^69^ IEC contact with microbial antigens and can also function in a similar way to immune cells. These cells express pattern recognition receptors (PRR), both toll-like receptors (TLR) and nucleotide-binding and oligomerisation domain (NOD)-like receptors (NLR), specifically NOD2, which can interact with pathogen-associated molecular patterns (PAMPs) and microbe-associated molecular patterns (MAMPs), derived from gut microbes. This elicits subsequent intracellular signaling via the NOD-like receptor family and pyrin domain-containing protein (NLRP) cascade to activate innate immune responses characterized by secretion of cytokines, chemokines and antimicrobial peptides, such as α-defensins, RegIII and lysozymes.^18,65,66^ For example, NOD2 expression was found to be essential for ensuring intestinal homeostasis, by preventing an excessive inflammatory response and the production of RegIIIβ, along with expansion of commensal *Bacteroides vulgatus*.^70^ The IEC layer also contains Paneth cells, which can receive input from commensals and cytokine signaling and that, dependent on fucosyltransferase 2, can prevent pathogen invasion, particularly in the ileum. This is mediated by secretion of antimicrobial peptides and the provision of epithelial fucose as a carbon source for certain gut commensals.^71,72^ Scattered throughout the epithelial barrier are intraepithelial lymphocytes, which can express a variety of cytokines and display a memory phenotype, giving these cells a privileged proximity to microbial antigens and a readiness to activate an immune response.^73^

Beneath the IEC layer is the lamina propria, a layer of connective tissue that contains both myeloid and lymphoid cells.^65^ Dendritic cells (DC) and macrophages can sample antigens from the gut microbiota and present them to B and T cells, which can orchestrate a targeted adaptive immune response.^65^ Most importantly, DC can protrude beyond the IEC barrier and directly sense microbial antigens from the gut lumen.^74^ Interactions between DC and T follicular helper/T follicular regulatory cells occur in the Peyer’s patches, specifically in the B cell follicles, where B cell maturation takes place, by undergoing class switch recombination and somatic hypermutation to produce high affinity antibodies, specifically IgA.^75^ IgA secretion is crucial for the modulation of gut microbial composition and control commensal bacteria abundance.^76^ Moreover, IgA coating also plays a direct role in pathogen recognition and elimination.^77^

Other immune cell populations are also involved in promoting a healthy gut environment, in particular the T helper 17 (Th17) and regulatory T cell (Treg) populations, both of which can be derived from CD4+ intestinal T cells.^18^ Th17 cells are an essential part of the intestinal immune response, producing a range of pro-inflammatory cytokines when stimulated, while adopting a quiescent profile under homeostatic conditions.^78^ Activation and subsequent cytokine secretion by this population is dependent on microbiota composition, as demonstrated by comparing their profile in response to commensal versus pathogenic bacteria.^79^ Treg, on the other hand, express the transcription factor Foxp3 to mediate a series of suppressive mechanisms including inhibition of co-stimulation of T cell activation, depletion of IL-2 required for effector T cells, production of immunosuppressive cytokines IL-10, IL-35, TGF-β and granzyme secretion.^80^ Treg cells can regulate Th17 abundance and activation, as suggested by research showing that Treg dysfunction caused by MyD88 deficiency leads to increased Th17 and intestinal dysbiosis, highlighting the need to maintain a stable Th17/Treg balance.^81^ Microbiota metabolites also have an impact in regulating these populations. For example, SCFAs can modulate Treg function by inducing Foxp3 expression.^82^ Polysaccharide A produced by *Bacteroides fragilis* can also contribute to a tolerogenic environment by increasing Treg abundance, and a concomitant suppression of Th17.^83^ In addition, supplementation of this bacterium to GF mice was able to induce Treg cytokine production in the gut.^84^

Another cell population found in the lamina propria are innate lymphoid cells (ILCs), which can be divided into cytotoxic natural killer cells and non-cytotoxic ILC1, ILC2 and ILC3 subsets.^65^ These cells play a critical role in maintaining the composition of the gut microbiota as shown by bacterial proliferation in an ILC-deficient mouse model associated with loss of IL-22 signaling.^85^ ILC3 are also required to induce the intestinal elimination of T cells reactive to commensal antigens, acting in a manner similar to thymic selection.^86^

## 4. The role of BEVs in immune homeostasis

### 4.1. Microbiota-derived BEVs

There is a growing need for tools to modulate changes in the gut microbiota composition. Studying bacterial dynamics may provide alternative ways to address such imbalances from within. Commonly used approaches include the transfer of whole gut microbiota isolates from healthy individuals, i.e. fecal microbiota transplantation (FMT), or defined microbial consortia.^87,88^ Other approaches include prebiotic or probiotic supplementation and modulation with inactivated bacteria or bacterial-derived components or metabolites, known as postbiotics.^89^ Recent research has identified BEVs as a new candidate for regulating microbiome dynamics with potential clinical applications. In fact, BEVs are deemed as essential components of microbial interactions in the context of the human microbiota, which until recently did not receive the deserved attention.^6,7,90^ Due to their small size, these vesicles can easily diffuse across the environment and target cells located far away from the parent bacteria. In fact, BEVs have recently been proposed as an independent type of secretion system, called type-0, capable of delivering a variety of cargoes, including virulence factors, nucleic acids, lipids and various proteins, which highlights their importance in the study of various diseases and conditions (Table 1).^91^ Studies have shown that specific effectors delivered by BEVs have a greater effect than when they are delivery freely to the gut lumen.^92^ Specific cargo composition relates to their potential to modulate gut microbial composition by stimulating cooperation or competition between different bacteria. For example, BEVs can carry polysaccharide-degrading enzymes that can contribute to the nutritional support of the producing strain, but also of other bacteria with the same nutritional requirements.^37^ Another study found that *Cupriavidus necator* can secrete the LPS-binding effector TeoL to recruit BEVs produced by different species and thus exploit their contents.^93^ This could include iron acquisition, nutrient-degrading enzymes, antibiotic resistance genes or other cargoes. Proteomic characterization of BEVs has been carried out on a variety of enteric pathogens and also on commensals, including several strains often used as probiotics.^46,94,95^ Similarly, other studies have included metabolomic and genomic characterization, particularly including RNA-seq experiments.^96^ Their specific RNA cargo, especially small non-coding RNAs, can interact with mRNAs in the target cells, including eukaryotic cells.^97^ This can lead to epigenomic changes, particularly in histone regulation.^98^ As well as enabling bacterial communication and delivery of active compounds between cells, BEVs can also potentially target invading microorganisms, maintaining populational balance and preventing external colonization or pathogen overgrowth.^99,100^

**Table 1.** Microbiota-derived BEVs and implications for specific disease models.

Bacteria	Model/disease	Findings	References
*Acinetobacter baumanii*	N/A	Provide iron delivery system for competition	145
*Akkermansia muciniphila*	Type 2 diabetes patients	BEVs abundance decreased in patients	14
	T2D mouse model	Improved gut permeability and disease scores	
	Colitis mouse model	Rescue of pathology features	127
*Bacteroides thetaiotaomicron*	Colitis mouse model	Symptoms improvement	11
	Murine bone marrow derived monocytes	Histone methylation changes	
	N/A	Glycosidases and proteases supply for competition	37
*Cupriavidus necator*	N/A	TeoL secretion to exploit other BEVs contents	93
*Faecalibacterium prausnitzii*	Colitis mouse model	Reduced inflammation and permeability	150
*Lactobacillus acidophilus*	N/A	Bacteriocin production against *Lactobacillus delbrueckii*	153
*Lactobacillus crispatus* and *L. gasseri*	Ex-vivo HIV-1 challenge	Decreased viral entry in cells	171
*Lactobacillus paracasei*	Colitis mouse model	Reduction of gut inflammation	12
*Lactobacillus plantarum*	Dermatitis mouse model (*S. aureus*)	Reduced atopic dermatitis features	166
	Vancomycin-resistant Enterococcus *C. elegans* model	Increased transcription of host defense genes	167
*Staphylococcus hominis*	*S. aureus* skin abscess mouse model	Micrococcin P1-dependent anti-*S. aureus* effect	152
*Vibrio cholerae*	*V. cholerae* infection	Toxin delivery to host cells	163
Various	IBD Chemotherapy-induced intestinal mucositis	LPS-positive BEVs detected in plasma samples	105
	Human lung microvascular endothelial cells	Endothelial barrier dysfunction	177

BEVs production by the gut microbiota has been linked to several diseases. Chelakkot and coworkers found a decreased abundance of BEVs derived from mucin-degrading *Akkermansia muciniphila* in patients with type 2 Diabetes (T2D), compared to healthy individuals, although no significant differences were observed in fecal *A. muciniphila* abundance.^14^ The reason behind a decreased abundance of *A. muciniphila* BEVs being implicated in disease pathogenesis can be because of cross-feeding interactions, since mucus degradation can provide nutritional support for other beneficial bacteria, such as butyrate-producing Clostridia, that consequently might also have been affected in this model and explain disease progression.^101^ In addition, *A. muciniphila* vesicles were found to improve gut permeability in a T2D mouse model, as measured by decreased serum FITC levels, and to rescue colon length and expression of occludin, a tight junction protein, highlighting the role of BEVs in mediating the function of bacteria involved in maintaining intestinal barrier function. The study also found an improvement in TD2-associated metabolic parameters.^14^ BEVs composition in the urine of patients with colorectal cancer^102^ and asthma^103^ showed significant differences compared to healthy controls. These studies suggest the use of urine analysis as a biomarker for disease, considering that BEVs can cross the intestinal barrier and enter systemic and lymphatic circulations.^104^ Indeed, LPS-positive BEVs have been detected in the plasma of patients with a compromised intestinal barrier, including those with IBD, radiation and chemotherapy-induced intestinal mucositis and HIV.^105^ Taken together, these findings underscore the importance of considering BEVs as a critical part of disease pathogenesis and therapeutic approaches.

### 4.2. BEVs and gut immune regulation

**Figure 4.**
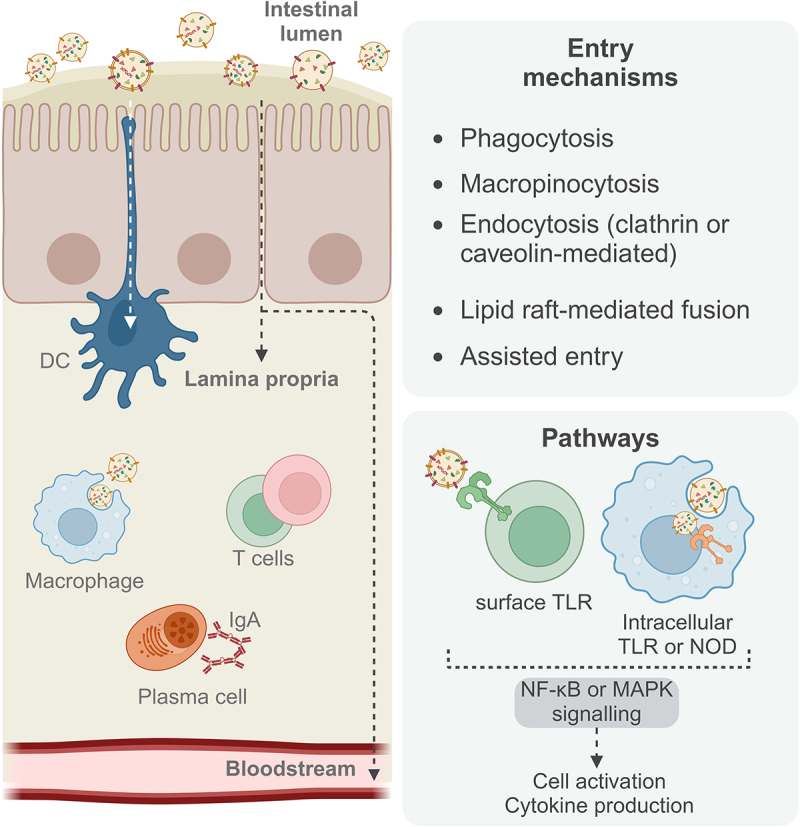
BEVs interactions with gut immune cells.

After leaving the parent bacteria, BEVs can directly entry IECs^106^ or alternatively cross the intestinal barrier by paracellular transport along the tight junctions^107^ and further interact with other immune and nonimmune cells in the lamina propria (Figure 4 and Table 2).^6,7,90^ This process was suggested by the observation that BEVs from *B. thetaiotaomicron* with sulphatase activity can be found inside mucosal macrophages,^108^ suggesting that vesicles can cross the intestinal barrier. In addition, BEVs can reach the bloodstream, which is related to the level of intestinal permeability and is therefore more prevalent in patients with colorectal cancer and colitis, but also in the elderly.^109^ BEVs can enter host cells by phagocytosis, which is the preferred route in DC and tissue macrophages, or by non-phagocytic mechanisms. These include macropinocytosis, clathrin-mediated or caveolin-mediated endocytosis (both dynamin-dependent) and lipid raft-mediated direct fusion or assisted entry.^9^ In addition, BEVs size appears to be a determinant factor in the type of host cell entry. A study using *Helicobacter pylori* BEVs showed that small vesicles tend to predominantly use the caveolin-mediated endocytosis pathway, in contrast to larger vesicles, that are mostly internalized by clathrin and dynamin-dependent endocytosis, evidenced using siRNA knockdowns of specific proteins from the different pathways.^110^ The same study also showed that vesicle size is positively correlated with protein cargo diversity.^110^

Bacterial extracellular vesicles present in the gut lumen can interact directly with intestinal epithelial cells, be phagocytosed by protruding dendritic cells, or alternatively cross the epithelial barrier and interact with lamina propria immune cells, including macrophages, T cells and B cells, using a variety of cell-type dependent entry mechanisms and activating specific signaling pathways to culminate in cell activation and cytokine secretion. BEVs can also exit the intestinal tissue via the bloodstream and disseminate systemically. (Created with BioRender.com).

**Table 2.** The role of BEVs in gut immune regulation.

Bacteria	Model/disease	Findings	References
*Aggregatibacter actinomycetemcomitans*	Human macrophage cell line (U937)	sRNA delivery via TLR8; NF-κB signaling; TNF-α release	125
*Akkermansia muciniphila*	*S. pullorum* chicken infection model	Commensal proliferation; DC and B cell activation; IgA increase; Goblet cell increase; Reduced permeability	149
*Bacteroides fragilis*	Colitis mouse model	TLR2-mediated dendritic cell entry; anti-inflammatory signaling	106
*Bacteroides thetaiotaomicron*	Genetic colitis mouse model	Sulfatase-mediated macrophage entry	108
	IBD patient-derived dendritic cells	IL-10 release in colonic DC; IL-6 release and CD80 expression in blood-derived DC	115
	Colitis scRNAseq dataset	Cell-type dependent TLR signaling	111
	Colitis mouse model	Colon and spleen IL-10 production	11
*Bifidobacterium bifidum*	Monocyte-derived DC culture	Treg differentiation, IL-10 release	121
*Bifidobacterium longum*	Splenocyte and DC/T cell cultures	IL-10 secretion	45
*Clostridium butyricum*	Colitis mouse model	M2 macrophage polarization	10
*Clostridium difficile*	Caco-2 cell culture	Expression of IL-1β, IL-6 and IL-8	118
*E. coli* (commensal)	Caco-2 and HT-29 cell cultures	NOD signaling	112
	Caco-2 and HT-29 cell cultures	Dose-dependent IL-8 release	114
	Human monocyte derived-DCs	DC cytokine secretion	120
	DC/T cell co-cultures	Polarization of naïve CD4 T cells	
	Caco-2 and PBMCs cultures	Epithelial barrier signaling	154
	*Ex-vivo* colon culture	Induction of cytokines and antimicrobial peptides	
*Fusobacterium nucleatum*	Macrophage/Caco-2 co-culture	Activation of RIPK1 cell death pathway	117
*Helicobacter pylori*	AGS and HEK293 cell lines	Size-dependent cell entry pathways	110
*Lactobacillus paracasei*	Murine macrophages and human colorectal cancer cells	Decrease in IL-1α, IL-1β, IL-2 and TNF-α; Increase in IL-10 and TGFβ	12
	Colitis mouse model	Rescue of disease features	
*Lactobacillus sakei*	Culture of Peyer’s patches cells	TLR-2 dependent IgA secretion	122
*Pseudomonas aeruginosa*	Lung epithelial cells	DNA delivery into host cells	32
	Primary airway epithelial culture	Rescued LPS-induced IL-8 secretion	126
Uropathogenic *E. coli*	Bladder epithelial cell culture	RNA delivery into host cells	124
*V. cholerae*	Epithelial cell culture and EC/DC co-culture	NOD signaling	113
Various	Colorectal cancer and colitis patients	BEVs in the bloodstream (intestinal permeability)	109
	Bone marrow-derived macrophages	Induction of mitochondrial apoptosis and NLRP3 inflammasome	119

In addition to interacting with intracellular pathways upon internalization, other BEVs simply interact with extracellular receptors to activate their immunomodulatory function. BEVs express various MAMPs, derived from the parent bacteria, that can activate PRR in host cells and intracellular signaling leading to immune activation. Specifically, BEVs interact with surface TLRs or, in the case of cell entry, with intracellular TLRs and NLRs, in particular NOD1 and NOD2. This leads to activation of the NF-κB or MAPK pathway, activating the transcription of inflammatory genes, followed by cell activation and cytokine production.^6^ For example, *Bacteroides fragilis* BEVs enter the host DC through an interaction between polysaccharide A on the vesicle surface and TLR2. This recognition is required to activate intracellular signaling to promote an anti-inflammatory response, which is implicated in the prevention of colitis.^106^ Bioinformatic analysis of *B. thetaiotaomicron* BEVs showed that they can target host cells differently and with contrasting consequences in terms of interaction with host cell proteins. These changes in protein-protein interactions and TLR signaling were dependent on cell type and the presence or absence of disease, particularly UC.^111^ Other TLRs can also be activated depending on the specific molecule in the BEVs that interacts with the receptor.^7^ NOD receptors can be activated by peptidoglycan contained in BEVs after internalization, as shown for two strains of commensal *E. coli* ^112^ and for the pathogen *V. cholerae*.^113^

The physiological consequences of these interactions may either promote tissue homeostasis by ensuring immune regulation or alternatively, be used to activate a targeted immune response when BEVs belong to a recognized pathogen.^7^ Indeed, BEVs derived from commensal *E. coli* C25 were associated to a dose-dependent release of IL-8 in a cell culture model of IEC.^114^ Furthermore, the study found that pre-incubation with BEVs inhibited internalization of the parent bacteria, suggesting that commensal signaling via BEVs could be used to fine-tune immune responses tailored to maintain tissue homeostasis.^114^ DC can also be regulated by BEVs, as shown by *B. thetaiotaomicron* BEVs which were able to induce IL-10 expression in colonic DC and IL-6 and CD80 expression in blood-derived DC,^115^ possibly through miRNA regulation.^116^ This system can and is exploited by pathogens to ensure their colonization, using a variety of mechanisms ranging from the induction of oxidative stress causing epithelial cell necroptosis,^117^ to the production of pro-inflammatory cytokines inducing intestinal epithelial cytotoxicity^118^ as well as mitochondrial dysfunction and subsequent inflammasome activation in macrophages leading to apoptosis.^119^

BEVs can be used to specifically regulate immune cell populations in the gut, namely by affecting the Th17/Treg balance and their cytokine secretion profile. Díaz-Garrido and colleagues found that BEVs released from different *E. coli* strains differentially modulate DC cytokine secretion to induce naïve CD4+ T cells to polarize in different directions, and that this is partly mediated by miRNAs.^120^ Specifically, the probiotic strain Nissle 1917 leads to increased secretion of IFN-γ and IL-12, associated with Th1 differentiation. The commensal strain ECOR12 induced secretion of TGFβ, necessary for Treg development.^120^ Similarly, a strain of *Bifidobacterium bifidum* was found to produce BEVs capable of promoting the differentiation of functional Treg and the release of the anti-inflammatory cytokine IL-10.^121^ Regarding the Th2 and Th17 populations, the authors found no strain-specific induction.^120^ Another study using BEVs from *Lactobacillus paracasei* found that they were able to rescue the cytokine profile induced by *in vitro* LPS administration, as confirmed by a decrease in the pro-inflammatory cytokines IL-1α, IL-1β, IL-2 and TNF-α and an increase in the anti-inflammatory cytokines IL-10 and TGFβ after BEVs treatment.^12^ Oral administration of the vesicles to a mouse model of colitis also protected against disease.^12^ Alternatively, pathogenic bacteria-derived BEVs were found to enhance inflammation, specifically by pyroptosis and subsequent NLRP3-mediated IL-1β release by macrophages, but also by inducing the loss of mitochondria function and consequently cell apoptosis.^119^ In addition, BEVs can also affect B cell immune responses by stimulating IgA secretion, as observed in a culture of Peyer’s patches cells, an effect that was abolished by inhibition of TLR2.^122^ Another method used by BEVs to interact with host cells is via nucleic acid delivery, both using bacterial DNA and small RNAs (sRNA), consisting of tRNA fragments.^123^ For example, *P. aeruginosa* BEVs have been found to deliver DNA into host cells, suggesting a possible mechanism of microbial regulation.^32^ Similarly, Blenkiron and colleagues showed that BEVs derived from uropathogenic *E. coli* can deliver a variety of RNAs, including protein-coding mRNAs into both the cytoplasm and nucleus of host cells.^124^ Once inside the cell, these RNAs can interact with host gene regulation.^125^ In the case of BEVs produced by *Aggregatibacter actinomycetemcomitans*, a bacterium involved in periodontal disease, sRNA delivered in these vesicles interacts with the intracellular TLR8, activates the NF-κB pathway and induces the production of TNF-α in a human macrophage-like cell line.^125^ Further research in an *in vivo* model using these BEVs also showed an increase in TNF-α production in the brain, suggesting that BEVs can cross the blood-brain barrier.^125^ Another *P. aeruginosa*-derived sRNA found in BEVs was able to reduce IL-8 secretion, previously induced by LPS treatment, in primary airway epithelial cells.^126^ Computational analysis identified human mRNA sequences to which this sRNA was complementary, highlighting the potential regulatory function that bacterial sRNA delivered by BEVs can have on host transcriptional regulation.^126^

BEVs modulation of intestinal immune responses has also been explored in the context of disease, particularly on gastrointestinal disorders, as evidenced by several studies showing the beneficial effect of specific bacteria-derived BEVs on colitis.^10–12,106,127^ For example, a study using a mouse model of colitis showed that the abundance of BEVs from different bacteria in the stool was altered after oral DSS administration. In particular, BEVs from *A. muciniphila* were found to be reduced. The oral administration of these vesicles to the mice showed a protective role on colitis pathology.^127^ BEVs from *B. thetaiotaomicron* also improved colitis symptoms in mice, associated with the promotion of a tolerogenic environment, characterized by increased IL-10 production in the colon and the spleen, possibly through interaction between BEVs and TLR2 on host cells, and through sustained changes in histone methylation patterns, as suggested by *in vitro* work.^11^ BEVs-mediated protection against colitis may also be related to their ability to regulate macrophage polarization, specifically by polarizing macrophages toward an M2 type, associated with tissue repair, as demonstrated with *Clostridium butyricum* BEVs.^10^

Taken together, these studies demonstrate how BEVs can stimulate immune cell activation and cytokine production, with implications for disease pathogenesis. However, to our knowledge, it remains to be demonstrated whether the reverse also occurs, i.e. that immune cells or specific cytokines stimulate BEVs secretion by members of the gut microbiota. This could potentially provide a strategy by which immune activation, either by pathogens or under basal homeostatic conditions, could help regulate the composition of the gut microbiome, in a feedback-based manner. Addressing this question will provide valuable insights into the mechanisms behind host-microbe interactions mediated by immune cells.

## 5. BEVs supplementation to reverse gut dysbiosis

### 5.1. Gut dysbiosis in disease

Imbalances in the composition and functional features of the gut microbiota are known as gut dysbiosis.^63,128^ Several factors can induce gut dysbiosis, including changes in lifestyle, such as physical activity and hygiene measures,^63,128^ diet,^129^ and environment,^130^ but also genetic background^131^ and clinical history, such as medication use, especially antibiotics,^61^ but also proton pump inhibitors, antidiabetics and antipsychotics.^132^

Changes in phylogenetic composition have been described in a variety of conditions, including gastrointestinal, metabolic, cardiovascular and neurodegenerative diseases.^50^ Mechanistically, microbiome dysbiosis can be caused by overgrowth of pathogens or pathobionts, loss of beneficial commensals or also by a substantial decrease in bacterial diversity.^63^ Diversity, richness and resilience have therefore been proposed as indicators of a healthy fecal material microbiome, and loss of these factors has been observed in a variety of diseases, such as IBD, AIDS and T1D.^63^ However, recent evidence has highlighted the relevance of the mucosal-associated microbiome as a better indicator of disease. For instance, in a mouse model of environmental Parkinson’s disease, the disease group had increased microbial diversity compared to the healthy group, based on ileum-associated bacteria.^133^ Some studies argue that a healthy gut microbiota can be defined by the Firmicutes/Bacteroidetes ratio, currently Bacillota/Bacteroidota, and that incremental or decremental deviations from a given value are associated with disease, particularly with obesity and IBD.^134^ However, further research has shown that these results should be interpreted with caution, as changes in this ratio do not necessarily correlate with disease^135^ and also change over a lifetime.^136^ These findings highlight the need to consider microbiota data at a much smaller scale, specifically at the family, genus or even species level whenever possible, where significant differences are often hidden.^137,138^

In terms of neurological diseases, a meta-analysis of the gut microbiome in Parkinson’s disease found an enrichment of *Lactobacillus*, *Akkermansia* and *Bifidobacterium* and a depletion of Lachnospiraceae and *Faecalibacterium* as common features between the different datasets analyzed.^139^ A similar approach also identified the predominant microbiome alterations in IBD, while presenting a powerful strategy for analyzing common microbiome signatures from patient cohorts of different diseases, leading to the definition of a universal dysbiosis index.^140^ In line with this, a previous meta-analysis highlighted disease-specific changes in the microbiome, compared to common changes, underlying the importance of careful extrapolation of findings from metagenomic studies.^141^ Significant changes at the species level can be masked by more widespread changes in composition, making it especially difficult to determine whether a particular strain or a small combination of strains is the real driver of disease onset. In addition, several researchers highlight the problem of dissecting whether changes in microbiota composition in a particular disease are a cause or rather a consequence of disease-related changes.^63^ Furthermore, and more importantly than specific compositional changes, recent research has pointed to the functional output of such altered dynamics as a more robust driver of disease-associated mechanisms. This includes the production of a range of metabolites that may have functional redundancy. Thus, abundance changes in different taxa can have the same functional consequences, and so explain inter-individual variation among patients with the same diagnosis. ^142–144^

Furthermore, gut dysbiosis is accompanied by dysregulation of the gut immune response, characterized by impairment of the intestinal barrier, together with dysregulated innate and adaptive immune responses, with a pronounced inflammatory profile.^63^ Taken together, the growing body of evidence on disease-associated changes in gut microbiota composition and immune profiles justifies the need for parallel development of powerful and efficient tools to modulate gut microbiota composition, taking into account the full extent of host-microbe interactions. In this context, and considering the number and diversity of microbial taxa in the gut, the pool of BEVs and their respective cargoes exponentially increases the complexity of host-microbe interactions, thus highlighting the need to consider BEVs in prospective research on gut dysbiosis, along with rationalizing strategies to restore healthy gut functions.

### 5.2. BEVs as a therapeutic strategy to fight gut dysbiosis

BEVs can be produced by gut commensals to regulate the composition of the microbiota and keep it within homeostatic parameters.^6,7^ This can be described as a natural phenomenon that should occur without disturbance in a healthy state. However, when perturbed, the resulting members of the commensal microbiota may not be able to provide this protective mechanism, allowing the development of gut dysbiosis and its potential pathogenic consequences.^63,128^ In addition, it can be assumed that invading bacteria or pathobiont blooms can lead to an increased production of harmful BEVs, culminating in the same harmful phenotype. Therefore, external manipulation with beneficial BEVs may be considered as a promising therapeutic intervention to promote the restoration of healthy commensal interactions and a reduction in pathogenic strains by exploiting their multiple mechanisms of interaction with the host and with other gut microbes.

The use of BEVs offers a number of advantages over other common methods of microbiota modulation. For example, BEVs are simple in structure and composition, with effects that are relatively easy to predict,^3^ in contrast to FMT, defined consortium transplantation, or even the addition of a single species, which can interact in a much wider variety of mechanisms that are difficult to manage given the complexity of the microbiome.^87,88^ Another advantage of BEVs therapy is their ability to retain active biological function and interact with a variety of targets depending on their cargo, which can be manipulated by bioengineering techniques to specifically load the vesicles with DNA, RNA or proteins of choice.^3^ This has another advantage over prebiotics, the only other method that is easy to characterize.^87^ Their stability and ability act as a vehicle for long distance communication in the body, especially when able to access the bloodstream, give them additional properties that support their use in the clinical setting to target gut dysbiosis.^3^ BEVs secretion can also be induced by a number of factors exposed above, which can be used to ensure high-yield production.^3,28^ Further understanding of these factors is essential to guide therapeutic approaches using BEVs, both as a natural stimulant of vesicle secretion *in vivo* as a therapy, and in the manufacturing process prior to clinical application. However, these factors should also be monitored as manipulation affects not only the rate of vesicle secretion but also their cargo.

**Figure 5.**
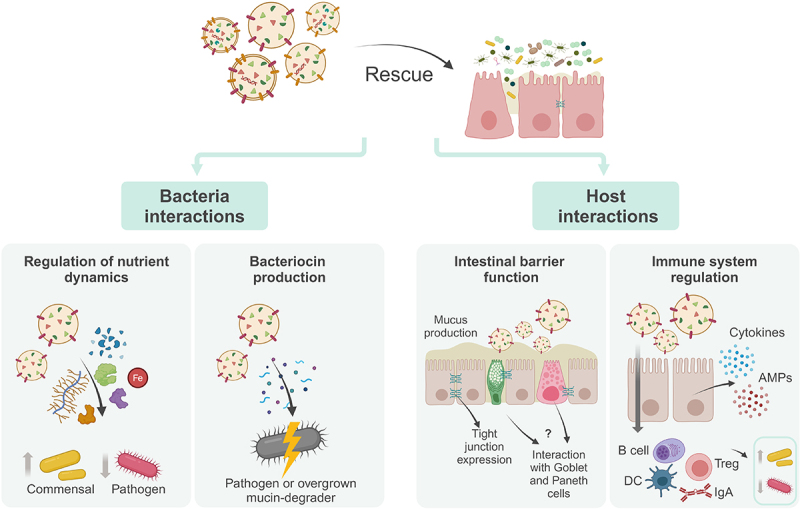
Potential of BEVs to rescue gut dysbiosis.

Mechanistically, BEVs can be used to combat gut dysbiosis in several ways (Figure 5). One way may be through direct interaction with other bacteria to promote commensal growth or, alternatively, pathogen restriction. By providing nutrient-degrading enzymes, such as glycosidases and proteases detected in *B. thetaiotaomicron* BEVs^37^ and also by acting as an iron delivery system, for example in *A. baumannii*,^145^ BEVs can be considered as a “public good” to mediate the nutrient supply of other commensals. Accordingly, therapeutic supplementation with BEVs could help regulate these interactions. Recent studies on the nutrient preferences of different gut commensals may support the production of BEVs containing the specific enzymes involved in the production of these key nutrients to sustain the growth of damaged populations.^146,147^ However, knowledge of commensal nutrient profiles still needs to be expanded.

Bacterial extracellular vesicles have the potential to restore healthy gut function and composition by interactions with gut bacteria and host functions. Interactions with bacteria include interference with nutrient dynamics and bacteriocin production, aiming to reestablish commensal dynamics and controlling pathogen proliferation. Host interactions include delivery of effectors that restore barrier function and regulation of gut immune dynamics, including by activating antimicrobial activity. DC, dendritic cell; Treg, regulatory T cell; AMPs, antimicrobial peptides. (Created with BioRender.com).

BEVs can also be used to stimulate intestinal barrier function, for example by promoting the growth of commensal *E. coli* and *A. muciniphila*, both of which are able to induce the expression of tight junction proteins, such as ZO-1, ZO-2 and claudin-14.^14,148^ A study in poultry found that *A. muciniphila* was able to rescue mucosal damage and was associated with an increase in goblet cell numbers, along with up-regulation of Mucin 2.^149^ Moreover, *B. thetaiotaomicron* was also found to stimulate upregulation of mucus-related genes in goblet cells, and these effects were reduced by co-administration of *F. prausnitzii*.^150^ However, it remains to be determined whether this interaction is mediated by BEVs. In another study in mice, *A. muciniphila* was able to promote the proliferation of intestinal stem cells as well as the differentiation of Paneth and goblet cells.^151^ Therefore, a viable approach could be based on the addition of vesicles capable of directly stimulating mucin production, by delivering the effectors produced by these strains. Alternatively, in patients with severe barrier erosion, mucin-carrying BEVs could be administered to provide a nutrient substrate for mucin-degrading bacteria, thus allowing the mucus layer to return to its normal thickness and seal the intestinal barrier. Delivery of BEVs-bearing bacteriocins targeting mucin degraders could be used to temporarily reduce the population and similarly allow recovery. Studies supporting bacteriocin-mediated inhibition by BEVs include a recent paper describing how the hydrophobic bacteriocin micrococcin P1 produced by commensal *Staphylococcus hominis* can be packaged into BEVs and fuse with *Staphylococcus aureus* to exert its effect, which was stronger compared to the same bacteriocin without association with BEVs.^152^ Dean and colleagues also showed that commensal *Lactobacillus acidophilus* BEVs could be enriched with bacteriocins after operon induction, which could then target the opportunist *Lactobacillus delbrueckii*.^153^

BEVs may indirectly promote intestinal barrier integrity by interacting with the mucosal immune system. Using a co-culture of the intestinal cell line Caco-2 and peripheral blood mononuclear cells (PBMCs) to replicate the intestinal mucosa, Fábrega and coworkers found that BEVs from probiotic and commensal *E. coli* were able to mediate signaling events through the intestinal epithelial barrier.^154^ These vesicles also induced cytokine and antimicrobial peptide production in an *ex-vivo* model of colon organ culture.^154^ In addition, in a cell culture model, *L. paracasei* BEVs were shown to reduce the release of TNF-α,^12^ a pro-inflammatory cytokine that is directly involved in intestinal barrier dysfunction.^155^ Other pro-inflammatory cytokines released by activated immune cells, such as IL-1β and IFN-γ, may also contribute to intestinal permeability.^156,157^ Thus, modulation of cytokine release by BEVs via interaction with immune cells may also be a viable method to restore intestinal barrier dysfunction and help recover gut homeostasis. Other interactions with immune cells to restore barrier loss can be achieved by stimulating the release of anti-inflammatory cytokines from Treg or reducing the activation of pro-inflammatory Th17, which can also regulate barrier function.^83,120,121^

In addition to focusing on the gut barrier, other avenues of interaction between BEVs and the immune system should also be explored, considering that immune cells and their effectors can also contribute to the regulation of microbiota composition by directly interacting with bacteria.^65,66^ When immune tolerance to commensals is compromised, leading to a depletion of beneficial strains, modulation by BEVs may help to restore a tolerogenic environment, allowing commensals to proliferate. An important consequence of gut dysbiosis is the subsequent immune dysfunction.^63^ Accordingly, acting directly on immune cells to reverse dysbiosis is another plausible method. To this end, we should focus on the ability of specific BEVs to modulate different immune populations, namely Tregs,^82,83,120,121^ DCs^120,121^ and also B cells to stimulate IgA production.^122^ Alterations in these populations have been well described in patients and in models of gut dysbiosis,^63^ highlighting the potential to use BEVs to regulate such responses.

Taken together, these strategies highlight the diversity of ways in which BEVs can be used to rescue gut microbiota dysbiosis, with potential benefits for the variety of diseases associated with this phenomenon. It is also important to note that more than one of these mechanisms may be used simultaneously by some bacteria. In fact, Wang and coworkers have recently shown that *A. muciniphila* BEVs is able to restore intestinal function through different mechanisms. These include stimulating commensal proliferation and increasing the richness and diversity of the microbiota; mediating immune cell activation, specifically DCs and B cells, together with a marked increase in intestinal IgA concentration; and promoting intestinal barrier function by increasing the number and function of goblet cells in the epithelium and the expression of the tight junction proteins ZO-1 and occludin in IEC.^158^

Most research to date has focused on the roles of BEVs produced by a single or a limited number of commensals at a time on gut microbiota dynamics. A comprehensive systematic analysis of the production of BEVs in the human gut, together with their associated effect on host health and disease, is only beginning to be explored. However, significant information on inter-individual variability in healthy and diseased individuals is already being collected.^159,160^ Furthermore, we hypothesize that similar to bacterial gut dysbiosis, BEVs dysbiosis may also be of fundamental importance for disease onset and progression, but also to guide therapeutic interventions based on the array of mechanisms outlined above.

### 5.3. BEVs for the management of colonisation resistance

In addition to gut dysbiosis involvement in the etiology of a number of inflammatory diseases, another important consequence of this phenomenon is the loss of colonization resistance to enteric pathogens, including to hospital-acquired infections.^161^ Colonization resistance consists in the ability of the gut microbiota to limit exogenous pathogen colonization or prevent pathobiont overgrowth, and is mediated by a number of direct and indirect mechanisms.^51^ Direct mechanisms include competition for available nutrients, often leading to the formation of specific nutritional and spatial niches occupied by commensals, resulting in nutrient depletion. Another method is the production of a variety of active molecules and metabolites that can act as bacteriocins to compete with other bacteria. These also include SCFAs, namely acetate, butyrate and propionate, and secondary bile acids, derived from primary bile acids that are not reabsorbed in the ileum. Bacteriophages also provide a form of direct protection by selectively targeting either a single bacterium or a small number of strains, thus helping to regulate the composition of the microbiota.^51,161^ Indirect mechanisms depend on oxygen availability, the presence of the inner and outer mucus layers that cover the intestinal epithelium to maintain barrier function, and interactions with the host innate and adaptive immune systems.^51,161^ This is demonstrated, for example, by the ability of LPS to stimulate TLR4 on IEC to prevent against vancomycin-resistant *Enterococcus* (VRE) colonization in mice, which is dependent on RegIIIγ expression in a MyD88-dependent manner.^162^

BEVs have a dual role in colonization resistance. Firstly, they can drive pathology by being produced by bacteria that are not abundant enough to cause disease, but with their long-range delivery of virulence factors to interact with a much wider range of host and microbial cells can produce pathology. A good example of the use of BEVs for pathogenesis has been observed in *V. cholerae*, which secrete BEVs with cholera toxin to enhance their ability to infect IEC.^163^ In addition, BEVs can contain DNA, which is another worrying finding given that they may be involved in lateral gene transfer between bacteria, including of antibiotic resistance genes.^164^ Some researchers have therefore suggested that some BEVs should be considered PAMPs, given their potential to activate inflammatory responses for example in the lung, specifically by inducing macrophage migration and increasing their phagocytic potential, together with the generation of reactive oxygen species, nitric oxide and cytokines.^165^ On the contrary, commensal-derived BEVs may have a protective role based on their interaction with other microbiota components and with host immune cells, providing an anti-inflammatory or a homeostatic inflammatory response. This may also help to prepare the immune system for a subsequent challenge with enteric pathogens. Kim and coworkers demonstrated a direct interaction between BEVs from a skin commensal and a pathogen, as the therapeutic administration of commensal *Lactobacillus plantarum* BEVs reversed atopic dermatitis features in a mouse model induced by administration of *S. aureus*-derived BEVs.^166^ Commensal-derived BEVs can also protect against pathogen colonization by directly stimulating host immune function. In a *C. elegans* model, *L. plantarum*-derived BEVs were shown to increase transcription of host defense genes and protect the nematodes from vancomycin-resistant Enterococcus colonization.^167^ The study also demonstrated vesicle uptake by the human intestinal cell line Caco-2, which was also associated with the transcription of defense genes, namely *CTSB* and *REG3G*.^167^ IL-22 secretion is also involved in ILC-mediated colonization resistance to *Citrobacter rodentium*, a rodent intestinal pathogen, in an ID2-dependent manner, an essential transcription factor for ILC3 function, as observed in mice lacking ID2.^168^

Remarkably, colonization resistance can also be mediated by viral pathogens. For example, the murine norovirus can provide colonization resistance against vancomycin-resistant Enterococcus by activating TLR7.^169^ *In vivo* work using a synthetic ligand for TLR7 in antibiotic-treated mice showed that this property is based on the restoration of REGIIIγ-mediated antimicrobial defense following IL-22 secretion by ILC.^169^ BEVs have been further proposed to protect against systemic viral infection by delivering DNA into host cells, leading to IFN-I priming.^170^ Another example of BEVs protection against viral infection is the inhibition of HIV-1 infection by lactobacilli BEVs derived from the vaginal microbiome of healthy women, assessed using human cell lines as a model.^171^ In addition, murine noroviruses have been shown to stimulate BEVs production by various gut commensals, providing a binding site for the virus and ultimately leading to an enhanced pro-inflammatory response by host cells, which recognize both the virus and the vesicles.^172^

The study of colonization resistance and the development of approaches aimed at preventing its loss or to restore healthy gut compositions are particularly relevant in light of the current threat of antimicrobial resistance (AMR). AMR is defined by the WHO as *one of the top global public health and development threats*, driven by the excessive and inappropriate use of antibiotics in human health but especially in animal production, and is estimated to be responsible for up to 10 million deaths per year by 2050.^173^ Antibiotic treatment is associated with the development of antimicrobial resistant bacteria, which can then share their antibiotic resistance with other members of the gut microbiota via a variety of mechanisms.^174,175^ This phenomenon exacerbates the increasing limitations of currently used antibiotics and highlights the need to find new and better alternatives to combat bacterial infections, especially antimicrobial resistant strains. In addition to the gut microbiota, the lung microbiota also shows an altered composition that predisposes to pathogen colonization. A recent study found that hospitalized patients with severe pneumonia had marked changes in specific genera, depending on the cause of the disease, either bacterial or viral.^176^ Given the importance of a protective microbiota in preventing lung diseases, such as hospital-acquired pneumonia, strategies to promote a healthy lung microbiome are urgently needed. Again, BEVs may have a pathogenic role, as evidenced by the observation that BEVs derived from sepsis-associated bacteria can potentiate their pathogenesis by inducing endothelial barrier dysfunction, specifically through TLR4 activation by LPS.^177^ Patients receiving mechanical ventilation also show a different BEVs composition on their plasma, when comparing pneumonia to non-pneumonia patients.^178^ In this setting, commensal-derived BEVs may have a beneficial effect, similar to that observed in the gut, in terms of promoting a tolerogenic immune landscape, characterized by an increased abundance of Treg cells and anti-inflammatory cytokines.^120,121^ Accordingly, new studies aimed at exploring the protective role of BEVs in lung immunity and resistance to infection are of great interest, potentially validating BEVs as a therapeutic agent to restore the lung microbiome dynamics.

BEVs modulation can be aimed to be used preventively, as an adjunct to antibiotic use, or as a tool to restore colonization resistance to enteric pathogens after a disruption. Furthermore, and according to this reasoning, BEVs can be used as a therapeutic method to ensure the maintenance of CR, for example to be used in hospitalized patients. We propose a dual mechanism of BEVs modulation for CR: positive and negative regulation. Positive regulation would be associated with promoting the growth of beneficial commensals through the various mechanisms highlighted in the previous section, whereas negative regulation would be associated with their concomitant ability to limit the growth of pathobionts and exogenous colonization. Together, positive and negative regulation could exploit the immunomodulatory role of BEVs, and guide the development of targeted interventions.

## 6. Conclusions and future perspectives

Despite some functional redundancy in the human gut microbiome,^142^ there is no single healthy microbiota composition^137,179^ nor does it remain unchanged over time.^180^ Moreover, an increased interest in the functional consequences of such compositional changes, motivated by the development of advanced metagenomic and metatranscriptomic technologies combined with hybridization methods and also single-cell analysis, has shed new light on the complexity of the mechanisms regulating the host-microbiome interplay.^66^ Considering the full extent of microbial interactions among themselves and with host cells, in particular IEC and cells of the innate and adaptive immune systems, is therefore crucial to fully understand how the gut microbiome regulates disease and to dissect the best strategies to modulate these dynamics.

Previous approaches have focused on modulating the composition of the microbiota through probiotic or prebiotic supplementation (combined are designated symbiotics) or alternatively, through fecal microbiota transplantation (FMT), currently only approved for the treatment of *Clostridioides difficile* infection but undergoing clinical trials for a variety of diseases ranging from IBD, irritable bowel syndrome and opportunistic gut infections, for example with carbapenemase-producing Enterobacterales, but also to autoimmune disorders, colorectal cancer, and neurodevelopmental and neurodegenerative disorders.^181^ However, given the limitations and potential side effects of this approach, such as gastrointestinal symptoms, the potential to introduce undetected pathogens or pathogenic compromise of beneficial commensal interactions, more precise methods are being explored.^87,88^ Recently, considering the potential of BEVs in the homeostatic modulation of gut composition via interspecies competition and also because of their role in stimulating the immune system, therapeutic approaches using these vehicles have attracted increasing attention.^3,6,7^ Indeed, a promising alternative approach to FMT would be explore the potential use of a fecal extracellular vesicle transplant (FET). This would provide a safer alternative, with detailed characterization and known targets, and most importantly that consists in a noninfectious bacterial product, though still able to stimulate protective immune dynamics. Moreover, the potential to engineer BEVs and to regulate their cargo composition and to specifically enrich them with selected compounds of interest highlights their role as promising therapeutic tools. Besides their potential application for regulating microbiota/immune interactions, BEVs are also currently being explored for a variety of other purposes, from vaccine production to drug delivery and as postbiotics.^3^ Despite their ease of large-scale production, changes in growth conditions can easily lead to variations in their cargo composition and compromise their clinical efficacy.^47,182^ Therefore, further research into their biogenesis regulation and cargo packaging mechanisms, as well as their stability, are relevant technical hurdles that need to be addressed and overcome before they can be used as therapeutic agents.

There is an urgent need to expand the characterization of vesicles from different gut bacteria, which is only just beginning, and to dissect their importance in each specific disease. New and exciting studies are being published every year, which together can provide more insight into this rapidly growing field by combining the efforts of immunology, microbiology and bioinformatics research, among others. Recent efforts have developed new tools to organize and provide easily accessible databases of BEVs research, including information on DNA, RNA, protein and lipid content.^183^ Metagenomic approaches have shown that changes in BEVs composition in stool samples can be a potential biomarker, especially considering that changes in BEVs can be more pronounced compared to changes in bacterial populations.^127^ In this review we have discussed microbiota-derived BEVs and their ability to regulate host intestinal immune responses. We have also discussed the potential of BEVs to modulate gut dysbiosis, by manipulating bacterial interactions in the gut and with immune cells. Given the diversity of diseases with microbiota dysbiosis and the opportunities this provides for pathogen colonization, further research should explore BEVs gut modulation in more detail. In particular, studies analyzing the entire BEVs pool in the gut microbiome, their cargo, interactions and effects are deemed important to provide a more complete understanding of BEVs biology and to guide future therapeutic approaches aimed at preventing or treating microbiome dysbiosis, not only in the gut, but also in other organs such as the skin or lungs.
